# Genome-wide analysis of copper, iron and zinc transporters in the arbuscular mycorrhizal fungus *Rhizophagus irregularis*

**DOI:** 10.3389/fpls.2014.00547

**Published:** 2014-10-14

**Authors:** Elisabeth Tamayo, Tamara Gómez-Gallego, Concepción Azcón-Aguilar, Nuria Ferrol

**Affiliations:** Departamento de Microbiología del Suelo y Sistemas Simbióticos, Estación Experimental del Zaidín, Consejo Superior de Investigaciones CientíficasGranada, Spain

**Keywords:** arbuscular mycorrhizal fungi, copper, iron, metal transporters, * Rhizophagus irregularis*, symbiosis, zinc

## Abstract

Arbuscular mycorrhizal fungi (AMF), belonging to the Glomeromycota, are soil microorganisms that establish mutualistic symbioses with the majority of higher plants. The efficient uptake of low mobility mineral nutrients by the fungal symbiont and their further transfer to the plant is a major feature of this symbiosis. Besides improving plant mineral nutrition, AMF can alleviate heavy metal toxicity to their host plants and are able to tolerate high metal concentrations in the soil. Nevertheless, we are far from understanding the key molecular determinants of metal homeostasis in these organisms. To get some insights into these mechanisms, a genome-wide analysis of Cu, Fe and Zn transporters was undertaken, making use of the recently published whole genome of the AMF *Rhizophagus irregularis*. This *in silico* analysis allowed identification of 30 open reading frames in the *R. irregularis* genome, which potentially encode metal transporters. Phylogenetic comparisons with the genomes of a set of reference fungi showed an expansion of some metal transporter families. Analysis of the published transcriptomic profiles of *R. irregularis* revealed that a set of genes were up-regulated in mycorrhizal roots compared to germinated spores and extraradical mycelium, which suggests that metals are important for plant colonization.

## INTRODUCTION

The transition metals Fe, Cu and Zn play essential and catalytic roles throughout the cell in various subcellular compartments. These metal cofactors are critical for processes such as transcription, translation, the production of ATP in the mitochondria and the scavenging of toxic free radicals (van Ho et al., 2002; Schaible and Kaufmann, 2004; Kim et al., 2008). However, these metals are a highly reactive group of elements and are toxic at high concentrations (Valko et al., 2005). Therefore, their biological concentrations are finely regulated in living cells. To maintain micronutrient homeostasis, all organisms have developed a complex network of metal uptake, chelation, trafficking and storage processes (Festa and Thiele, 2011). Transporters represent the first line of defense to perturbations of cellular and subcellular metal homeostasis and constitute an important component of this network. When metal reserves are depleted, transporters contribute to the specific supply and distribution of the needed cofactor before deficiency symptoms appear. However, when the concentration of metal within the cell exceeds the cell’s buffering capacity, transporters provide the route to expel excess cofactors before toxicity occurs (Nies, 2007). The toxic heavy metals, such as cadmium, lead, mercury, and nickel, have no physiological function but compete with the transporters of the essential biological metals. Therefore, the activity and specificity of the transporters of physiologically important heavy metals also control the lethality of the toxic metals.

Arbuscular mycorrhizal fungi (AMF) are soil microorganisms that establish symbiotic mutualistic associations with most land plants. These fungi provide their host plants an efficient supply of low mobility mineral nutrients, mainly phosphorus and some micronutrients such as Cu and Zn. Thanks to the hyphal network they develop in the soil, AMF acquire nutrients not only for their own needs, but also for delivering them to the host plant. In return, the plant supplies the fungus with carbon compounds (Smith and Read, 2008). Besides improving plant mineral nutrition, AMF can alleviate heavy metal toxicity to their host plants (Göhre and Paszkowski, 2006; Lingua et al., 2008). Heavy metal tolerant AMF ecotypes have been isolated from polluted soils and these indigenous populations cope better with heavy metal-toxicity than those isolated from unpolluted soils (del Val et al., 1999). To persist in environments with high heavy metal content, AMF have evolved a series of strategies to avoid the damage produced by the metal, such as compartmentalization of the metal excess in some spores (González-Guerrero et al., 2008; Cornejo et al., 2013) and highly efficient homeostatic mechanisms (Ferrol et al., 2009; González-Guerrero et al., 2009). Despite the central role of metal transporters in heavy metal homeostasis, only a gene encoding a Zn transporter has been characterized in AMF to date (González-Guerrero et al., 2005).

With the genome of *Rhizophagus irregularis* available (Tisserant et al., 2013), we have the unique opportunity to identify and present a global view of proteins involved in heavy metal transport in an AM fungus. In this work we have taken advantage of the recently released genome sequence of *R. irregularis* to establish a repertoire of candidate genes potentially involved in the transport of Cu, Fe and Zn in this fungus and to interpret them in light of its extremely adaptable character to grow in conditions of heavy metal deficiency or toxicity. This *R. irregularis* repertoire has been compared with that present in some reference fungi. To get some clues about the expression profiles of these genes throughout the fungal life cycle, we explored the published transcriptomic profiles in the extraradical mycelium (ERM) and symbiotic roots (intraradical mycelium, IRM) obtained using the *R. irregularis* expression oligoarray (Tisserant et al., 2012) and the RNA-Seq reads obtained from germinated spores and *Medicago*-colonized roots (Tisserant et al., 2013).

## MATERIALS AND METHODS

### GENE IDENTIFICATION

Amino acid sequences of fungal Cu, Fe, and Zn transporters were retrieved from the freely accessible transport databases TCDB^1^ and TransportDB^2^. These sequences were used to search for orthologous sequences in the filtered model dataset of *R. irregularis* on the JGI website^3^ via a protein BLAST. A second search was performed via a keyword search directly.

Since many of the fungal reference proteins were phylogenetically distant from *R. irregularis*, manually curated *Laccaria bicolor*^4^ *Tuber melanosporum*^5^ and *Rhizopus oryzae*^6^ databases were used to look for additional orthologous sequences in the filtered model dataset of *R. irregularis*. This was also done via a BLASTp, run with the standard program settings.

### SEQUENCES ANALYSES

Searches for conserved domains in the orthologous proteins found in *R. irregularis* were performed using the Conserved Domain Database at NCBI^7^. Predictions of putative transmembrane domains were made using the TMHMM Server v.2.0^8^ and SMART software^9^. Full-length amino acid sequences were aligned with the orthologous sequences of a number of fungi representatives of distinct taxonomic groups by CLUSTALW^10^. Alignments were imported into the Molecular Evolutionary Analysis (MEGA) package version 6. Phylogenetic analyses were performed using the Neighbor-Joining (NJ) method implemented in MEGA using the Poisson correction model and pairwise deletion of gaps option for distance computation. Bootstrap analyses were carried out with 1000 replicates.

## RESULTS AND DISCUSSION

The release of the *R. irregularis* genome (Tisserant et al., 2013; Lin et al., 2014) allowed making a genome-wide inventory of genes coding for Cu, Fe, and Zn transporters. This *in silico* analysis allowed identification of 30 open reading frames in the *R. irregularis* genome, which potentially encode heavy metal transporters. These candidate genes belong to several multigene families. **Table 1** lists the eight phylogenetic families to which these proteins belong and the major heavy metal substrate for each transporter. These heavy metal transport families are described in the following sections.

**Table 1:** Overview of the putative metal transporters identified in the *Rhizophagus irregularis* genome.

Protein name	JGI ID	Major substrate	Ratio IRM/spore	Ratio IRM/ERM
**CTR family**
CTR1	153709	Cu	0.5	–
CTR2	335281	Cu	5	2.9
CTR3	67076	Cu	0.3	–
**P_**1B**_-ATPase family**
CCC2.1	335789	Cu	2.6	2.6
CCC2.2	83433	Cu	1	–
CCC2.3	236684	Cu	0.7	–
CRD1	32309	Cu	1	–
**SIT family**
SIT1	305535	Sid.-Fe	0.6	–
SIT2	193231	Sid.-Fe	1.9	1.9
SIT3	71812	Sid.-Fe	1	–
**OFet family**
FTR1	347887	Fe	10	–
FTR2	34848	Fe	1.5	–
**VIT family**
CCC1.1	278480	Fe/Mn	0.9	3.4
CCC1.2	57183	Fe/Mn	0.04	0.1
CCC1.3	340222	Fe/Mn	2.7	–
**ZIP family**
ZRT1	327155	Zn	420	8
YKE4	337446	Zn	0.9	–
ATX2	80864	Mn	1.2	–
ZRT3.1	13899	Zn	3.5	–
ZRT3.2	336612	Zn	3	14
**CDF family**
ZnT1	70407	Zn	100	–
ZnT2	286233	Zn	1	–
MMT1	85722	Fe	2	–
MSC2	340453	Zn	0.9	0.9
ZRG17	67256	Zn	1	0.9
MnT1	232215	Mn	0.8	0.8
**NRAMP family**
SMF1	136431	Mn/Fe	3	0.9
SMF2	89717	Mn	6	–
SMF3.1	313253	Fe	1.9	–
SMF3.2	337501	Fe	0.7	1

*Columns 1–5 contain protein name, protein JGI identification (JGI ID) number, predicted major metal transported, ratio of expression levels in *M. truncatula* symbiotic roots (IRM) to 2 d germinated spores (calculated from the RNA-seq reads in Tisserant et al., 2013), and ratio of expression levels in IRM to ERM (from Tisserant et al., 2012). CTR, Cu transporter; SIT, siderophore-iron (Sid.-Fe) transporter; OFet, oxidase-dependent Fe^2+^ transporter; VIT, vacuolar iron transporter; ZIP, zinc-iron permease; CDF, cation diffusion facilitator; NRAMP, natural resistance-associated macrophage protein.*

### COPPER

**FIGURE 1:**
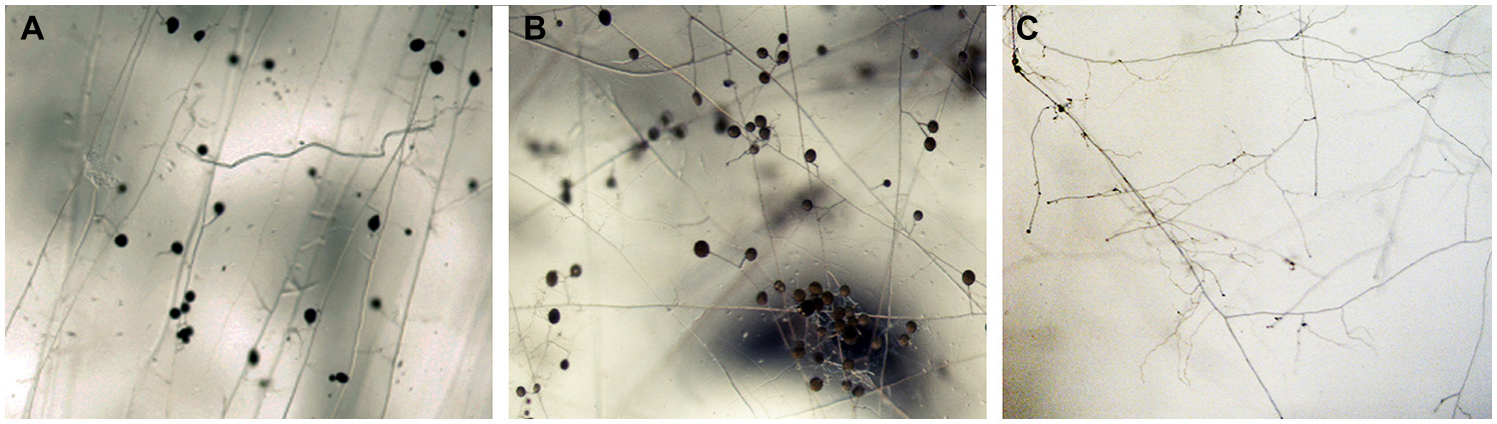
**Effect of Cu on extraradical hyphal development of *Rhizophagus irregularis* grown *in vitro* in association with root organ cultures.** Extraradical mycelium (ERM) was grown in minimal medium lacking Cu **(A)**, containing 0.5 μM Cu **(B),** or 500 μM Cu **(C)**. Mycelial architecture was altered markedly when the fungus developed in a Cu-free media **(A)** and in the presence of 500 μM Cu **(C)**.

Despite the long history of Cu as fungicide, AMF are able to grow and persist in Cu contaminated soils. The morphological alterations observed in the ERM of *R. irregularis* grown *in vitro* in association with root organ cultures in media without Cu or with Cu concentrations that are lethal to a majority of other organisms reflect its extremely adaptable character (**Figure 1**). Several studies have shown that AMF finely regulate the cytosolic Cu levels when confronted to excess Cu (González-Guerrero et al., 2008) and that the fungus responds to Cu toxicity by inactivating the excess of Cu in the cytosol through the activity of metallothioneins and the activation of antioxidant defenses (for a review see Ferrol et al., 2009). However, nothing is known about the Cu transporters that move Cu across the *R. irregularis* membranes. The two major families of Cu transporters identified in the *R. irregularis* genome are described below.

#### The copper transporter (CTR) family

Our *in silico* analysis revealed that *R. irregularis* likely acquires Cu through the activity of a transporter belonging to the CTR family of Cu transport proteins. This protein family is highly conserved across all fungal species and mediates Cu transport into the cytoplasm. CTR proteins are small integral membrane proteins that contain three transmembrane domains, with the N-terminus located in the extracellular space and the C-terminus in the cytosol. A series of clustered methionine residues in the hydrophilic extracellular domain, and a MXXXM motif in the second transmembrane domain, are important for Cu uptake. These methionine residues probably coordinate Cu during the process of metal transport (Yuan et al., 2011).

**FIGURE 2:**
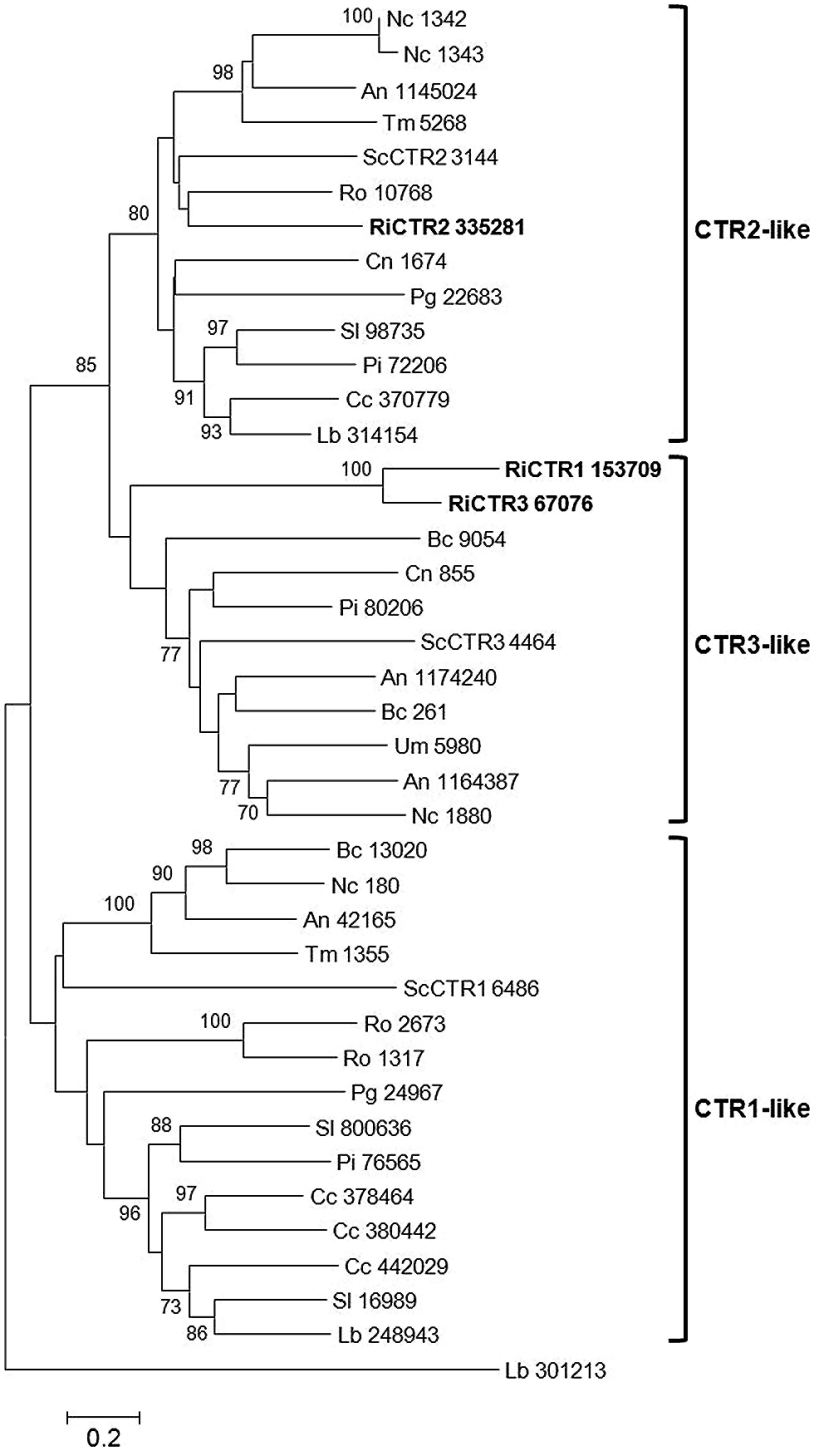
**Phylogenetic relationships of the *Rhizophagus irregularis* copper transporters (CTR) with homologous sequences from selected species representative of the major fungal phyla.** The Neighbor-Joining tree was created with MEGA 6. Protein JGI identification numbers are indicated. *R. irregularis* genes are shown in bold. Organisms: An, *Aspergillus niger*; Bc, *Botrytis cinerea*; Cc, *Coprinopsis cinerea*; Cn, *Cryptococcus neoformans*; Lb, *Laccaria bicolor*; Nc, *Neurospora crassa*; Pi, *Piriformospora indica*; Pg, *Puccinia graminis*; Sc, *Saccharomyces cerevisiae*; Sl, *Suillus luteus*; Tb, *Tuber melanosporum;* Ri, *Rhizophagus irregularis*; Ro, *Rhizopus oryzae*; Um, *Ustilago maydis*. Bootstrap values above 70 and supporting a node used to define a cluster are indicated.

In *Saccharomyces cerevisiae*, Cu is transported into the cytosol by three high-affinity transporters (CTR1, CTR2, and CTR3). While CTR1 and CTR3 are located in the plasma membrane, and acquire Cu from the environment (Dancis et al., 1994; Marjorette et al., 2000), CTR2 is found in the tonoplast and pumps Cu into the cytosol (Portnoy et al., 2001; Puig and Thiele, 2002). The *R. irregularis* genome also contains three genes putatively encoding CTRs. The predicted genes and proteins have been named according to their orthologs in *S. cerevisiae*. These proteins clustered into two different clades in a phylogenetic Neighbor-Joining tree. RiCTR1 and RiCTR3 are more closely related to the *S. cerevisiae* plasma membrane CTR proteins, while RiCTR2 is highly homologous to the fungal vacuolar CTR2 transporters (**Figure 2**).

Since CTR proteins are highly specific for reduced Cu^+^ and Cu widely exists as Cu^2+^, transport by CTR is dependent on reduction of Cu by a ferric/cupric reductase (Hassett and Kosman, 1995). Orthologous sequences of the fungal cell surface Cu metalloreductases encoded by the FRE genes are present in the *R. irregularis* genome (see next section), suggesting that the Cu reduction process is similar to that described for other fungi. Upon entering the cytoplasm, small molecules and proteins sequester the Cu ions, and the resulting concentration gradient drives transport by CTR.

Inspection of the available gene expression profiles of *R. irregularis* revealed that RiCTR2 is up-regulated in *Medicago truncatula* colonized roots, suggesting that some Cu is mobilized from the internal stores in the IRM probably to provide Cu to Cu-binding proteins that might be required for fungal colonization (**Table 1**).

#### Copper-transporting P-type ATPases

Copper-transporting ATPases belong to the heavy metal P-type ATPase family (HMA), also known as P_1B_-ATPases, which couples ATP hydrolysis to the eﬄux of positively charged metals from the cytoplasm. These proteins possess eight transmembrane domains, a large cytoplasmic loop, including ATP-binding and phosphorylation sites, and at least one conserved CPX motif (i.e., CPC) believed to be involved in metal cation translocation across the membrane.

**FIGURE 3:**
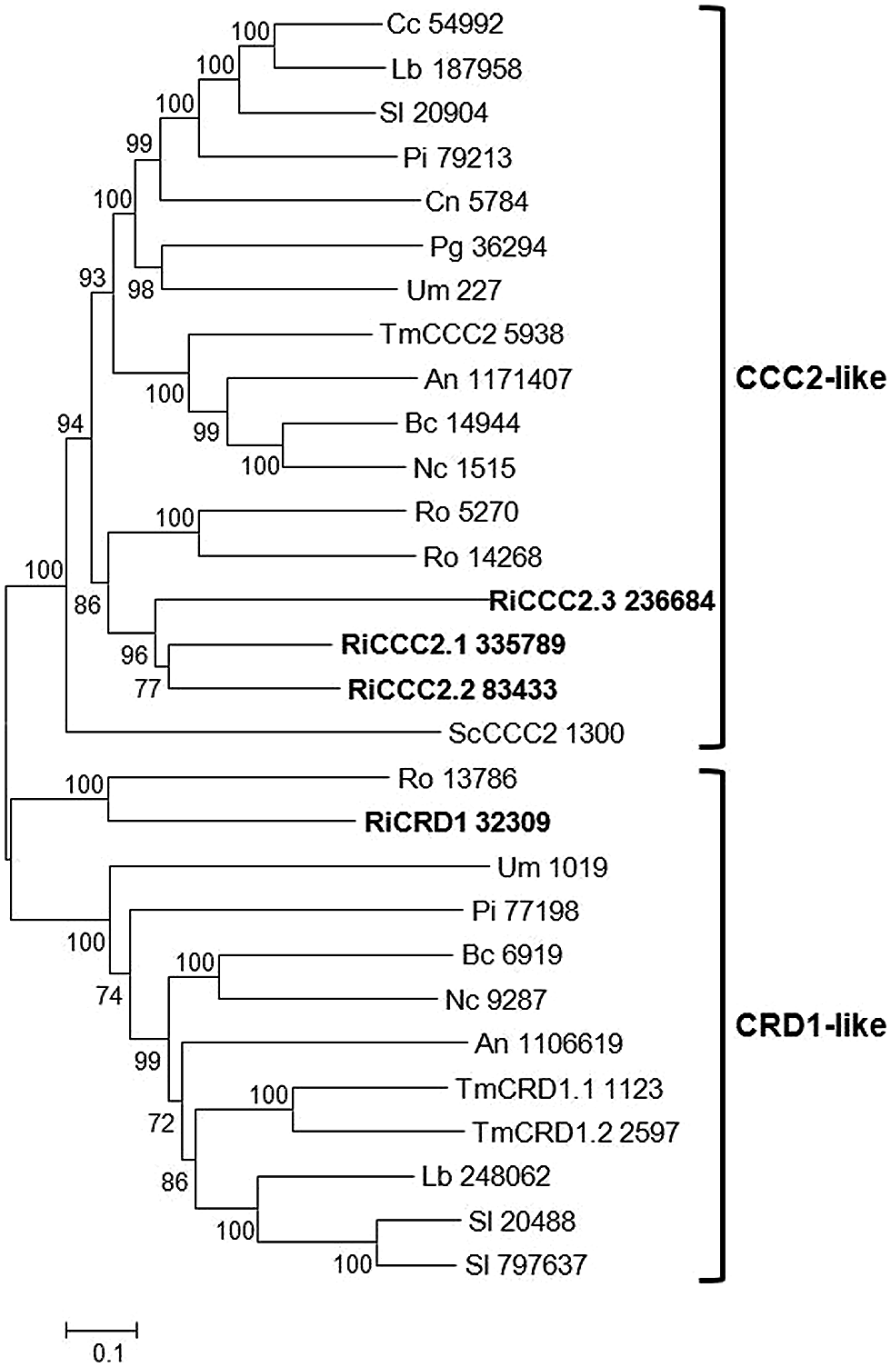
**Phylogenetic relationships of the *Rhizophagus irregularis* copper-transporting P-type ATPases (CCC2) with homologous sequences from selected species representative of the major fungal phyla.** The Neighbor-Joining tree was created with MEGA 6. Protein JGI identification numbers are indicated. *R. irregularis* genes are shown in bold. Organisms: An, *Aspergillus niger*; Bc, *Botrytis cinerea*; Cc, *Coprinopsis cinerea*; Cn, *Cryptococcus neoformans*; Lb, *Laccaria bicolor*; Nc, *Neurospora crassa*; Pi, *Piriformospora indica*; Pg, *Puccinia graminis*; Sc, *Saccharomyces cerevisiae*; Sl, *Suillus luteus*; Tb, *Tuber melanosporum;* Ri, *Rhizophagus irregularis*; Ro, *Rhizopus oryzae*; Um, *Ustilago maydis*. Bootstrap values above 70 and supporting a node used to define a cluster are indicated.

Four candidate genes putatively encoding Cu^+^-ATPases have been found in the *R. irregularis* genome, which represents an expansion compared with other fungi (). These genes were clustered in two different groups in a phylogenetic tree (**Figure 3**). Three of them were grouped in a clade comprising the well characterized ortholog of *S. cerevisiae* CCC2, a protein that receives Cu from the Cu chaperone ATX1 via a direct protein–protein interaction, and pumps Cu into the late- or post-Golgi compartment to load Cu into a multicopper oxidase required for Fe uptake (see next section) and, presumably, to other Cu-dependent proteins (Yuan et al., 1995). The other paralog, RiCRD1, groups in a different clade comprising various orthologs of CRD1, a plasma membrane Cu^+^-ATPase that plays a major role in Cu detoxification via Cu eﬄux in the opportunistic fungus *Candida albicans* (Weissman et al., 2000). Although it has been suggested that functions of the fungal Cu-ATPases can be inferred from their positions in a phylogenetic tree (Saitoh et al., 2009), a detailed characterization of the *R. irregularis* paralogs is needed to understand their physiological functions.

**Table 2:** Number and classification of the putative Cu, Fe, and Zn transporters identified in the genome of *R. irregularis* and in the genomes of the reference fungi used in this study.

	Basidiomycota							Ascomycota					Glomeromycota	Mucoromycotina
	*Puccinia graminis*	*Ustilago maydis*	*Laccaria bicolor*	*Coprinopsis cinerea*	*Cryptococcus neoformans*	*Piriformospora indica*	*Suillus luteus*	*Saccharomyces cerevisiae*	*Tuber melanosporum*	*Aspergillus niger*	*Neurospora crassa*	*Botrytis cinerea*	*Rhizophagus irregularis*	*Rhizopus oryzae*
OFet (Ftr1)	1	1	3	0	3	1	1	2	1	3	1	1	2	1
Low affinity Fe^2+^ transporter	0	0	0	0	0	0	0	1	0	1	0	1	0	0
NRAMP	3	1	1	1	1	0	2	3	0	1	2	1	4	4
ZIP	2	3	7	4	4	3	5	5	7	8	8	7	5	7
VIT	4	1	1	1	1	1	2	1	1	2	1	1	3	2
CTR	2	1	2	4	2	3	3	3	2	4	4	3	3	3
P_1B_-type ATPases	1	2	2	1	1	2	3	1	3	2	2	2	4	3
CDF	2	5	9	5	5	5	7	6	4	6	5	6	6	4

*CTR, Cu transporter; OFet, oxidase-dependent Fe^2+^ transporter; VIT, vacuolar iron transporter; ZIP, zinc-iron permease; CDF, cation diffusion facilitator; NRAMP, natural resistance-associated macrophage protein.*

Analysis of the available expression profiles of *R. irregularis* revealed a 2.6-fold up-regulation of RiCCC2.1 gene expression in mycorrhizal roots relative to the expression levels in spores and ERM. No data are still available of the expression profiles of the other paralogs in the ERM. Up-regulation of RiCCC2.1 in the symbiotic stage, as it has been observed for the Cu transporter RiCTR2, suggests a role for these proteins to supply Cu to other enzymes required for fungal accomodation or functioning in the root tissues. In this respect, it has been shown that the CTR2 ortholog of the plant pathogen *Colletotrichum gloeosporioides* (Barhoom et al., 2008) and the CCC2 orthologs of *Colletotrichum lindemuthianum* (Parisot et al., 2002) and *Botrytis cinerea* (Saitoh et al., 2010) are required for pathogenicity.

### IRON

#### Iron uptake

Although Fe is abundant in nature, this metal has a low availability because it is most commonly found as ferric hydroxide, which is a rather stable and poorly soluble compound. A common strategy engaged by fungi to efficiently get the metal involves “sequestering” of Fe through the production and subsequent uptake of siderophores, which are small molecules that act as high-affinity Fe chelators (Haas et al., 2008). Another important Fe uptake mechanism involves a group of specialized membrane proteins that are part of the reductive iron assimilation system (RIA). In this high-affinity uptake machinery, the metal is reduced from Fe^3+^ to Fe^2+^ (in order to increase Fe solubility) by membrane-bound ferrireductases, and then it is rapidly internalized by the concerted action of a ferroxidase and a permease that form a plasma membrane protein complex (Kosman, 2010).

A number of fungi harbor both types of high affinity systems, examples are *Ustilago maydis*, *Schizosaccharomyces pombe*, *Aspergillus fumigatus* and *Fusarium graminearum* (Mei et al., 1993; Roman et al., 1993; Askwith and Kaplan, 1997; Schrettl et al., 2004; Eichhorn et al., 2006; Schwecke et al., 2006; Greenshields et al., 2007). Others, such as *S. cerevisiae*, *C. albicans* and *Cryptococcus neoformans* (Schwyn and Neilands, 1987; Lesuisse and Labbe, 1989; Howard, 1999), are unable to synthesize siderophores but can utilize those produced by other organisms.

#### The siderophore pathway

Arbuscular mycorrhizal fungi are assumed to play a key role in Fe uptake and delivery to their associated host plants. However, it is still unknown whether AMF produce siderophores. The majority of fungal siderophores are hydroxamates. The first committed step in the biosynthesis of fungal hydroxamate siderophores is the *N5*-hydroxylation of ornithine catalyzed by ornithine-*N5-*monooxygenase (named Sid1/SidA). The absence of a Sid1/SidA ortholog in a fungal genome is generally taken as a strong evidence of no siderophore production. Inspection of the *R. irregularis* genome indicates that it does not contain Sid1/SidA orthologs. Similarly, the genomes of Saccharomycotina and Mucoromycotina and some Basidiomycota lack genes coding for this enzyme, which is in agreement with the observed lack of siderophore production by these fungi (Lesuisse and Labbe, 1989; Plattner and Diekmann, 1994). Interestingly, although the *L. bicolor* genome also lacks Sid1/SidA orthologous genes, production of a set of different hydroxamate siderophores by *L. bicolor* has been recently reported (Haselwandter et al., 2013).

A gene encoding a putative bifunctional iucA/iucC siderophore biosynthetic protein (RiSid1) that is highly expressed in mycorrhizal roots was found in the genome of *R. irregularis*. IucA and iucC catalyse discrete steps in the biosynthesis of the siderophore aerobactin from N epsilon-acetyl-N epsilon-hydroxylysine and citrate. The C-terminal region of RiSid1 is related to the bacterial ferric iron reductase FhuF-like transporter. The genomes of the reference fungi used in this study also contain orthologs of this gene. Therefore, the production of siderophores by *R. irregularis* remains uncertain.

Irrespective of their ability to produce siderophores, fungi have siderophore transporters that allow them to uptake different types of these small chelators, including bacterial ones like coprogen or enterobactin. This allows several fungi to take advantage of the siderophores produced by other organisms, securing in such manner their own iron needs (Haas et al., 2008; Saha et al., 2013). This type of siderophore transporters belong to the SIT (siderophore-iron transporter) subfamily (2.A.1.16) of the major facilitator superfamily, a protein subfamily present exclusively in fungi (and not in other eukaryotes or prokaryotes). SITs are secondary transporters with 12–14 predicted transmembrane domains, which likely function as proton symporters energized by the plasma membrane potential (Haas et al., 2003; Philpott and Protchenko, 2008). Searches in the *R. irregularis* genome using as query the SIT genes of *S. cerevisiae* and *S. pombe* allowed identification of three putative siderophore transporters (RiSIT1, RiSIT2, and RirSIT3), which are expressed in all fungal structures (**Table 1**). Detailed characterization of these transporters is needed to determine their substrate specificity.

#### The reductive iron assimilation (RIA) pathway

The reductive iron assimilation pathway starts with reduction of ferric iron sources to the more soluble ferrous iron Fe^2+^ by plasma membrane-localized ferrireductases. *R. irregularis* contains a putative ferrireductase (RiFRE1) that displays the highest homology to the *S. cerevisiae* FRE2 (23% identity and 43% similarity), a protein that can reduce oxidized forms of both Fe and Cu. RiFRE1 encodes a 541 amino acids protein that has seven transmembrane regions, several NAD(P)H binding motifs and a FAD binding motif.

Reduced Fe is then specifically taken up by a high-affinity transport complex consisting of a ferroxidase and a Fe permease, the oxidase-dependent Fe^2+^ transporter (OFeT), or non-specifically through other plasma membrane divalent cation transporters, as it will be discussed below. In *S. cerevisiae*, Fe^2+^ can be also taken up by the low-affinity Fe transporter Fet4 (Dix et al., 1994). This low-affinity system seems to be absent in *R. irregularis* as well as in the other reference fungi used in this study, except the ascomycetes *S. cerevisiae*, *A. niger,* and *B. cinerea* (**Table 2**).

#### The oxidase-dependent Fe^2+^transporter (OFeT) family

In *S. cerevisiae*, the ferroxidation/permeation pathway is mediated by the ferroxidase FET3 and the iron permease FTR1. This bipartite complex operates with an apparent *Km* of 0.2 μM. The Fe^2+^ to be transported is first oxidized by FET3, and then transported into the cytosol as Fe^3+^ by FTR1 via a channeling mechanism (Kwok et al., 2006). The advantage gained by redox coupling of this transport mechanism is unclear, although it possibly imparts specificity to transport. FET3 contains a single transmembrane domain and an extracellular multicopper oxidase domain, showing remarkable similarity to other multicopper oxidases, such as laccases and ascorbate oxidases. Searches in the *R. irregularis* genome for ferroxidases retrieved several genes putatively encoding multicopper oxidases (data not shown). The encoded proteins are more closely related to members of the ferroxidase/laccase subfamily of multicopper oxidases than to ferroxidases *sensu stricto*. Detailed characterization of these genes will enable identification of the FET3 ortholog.

**FIGURE 4:**
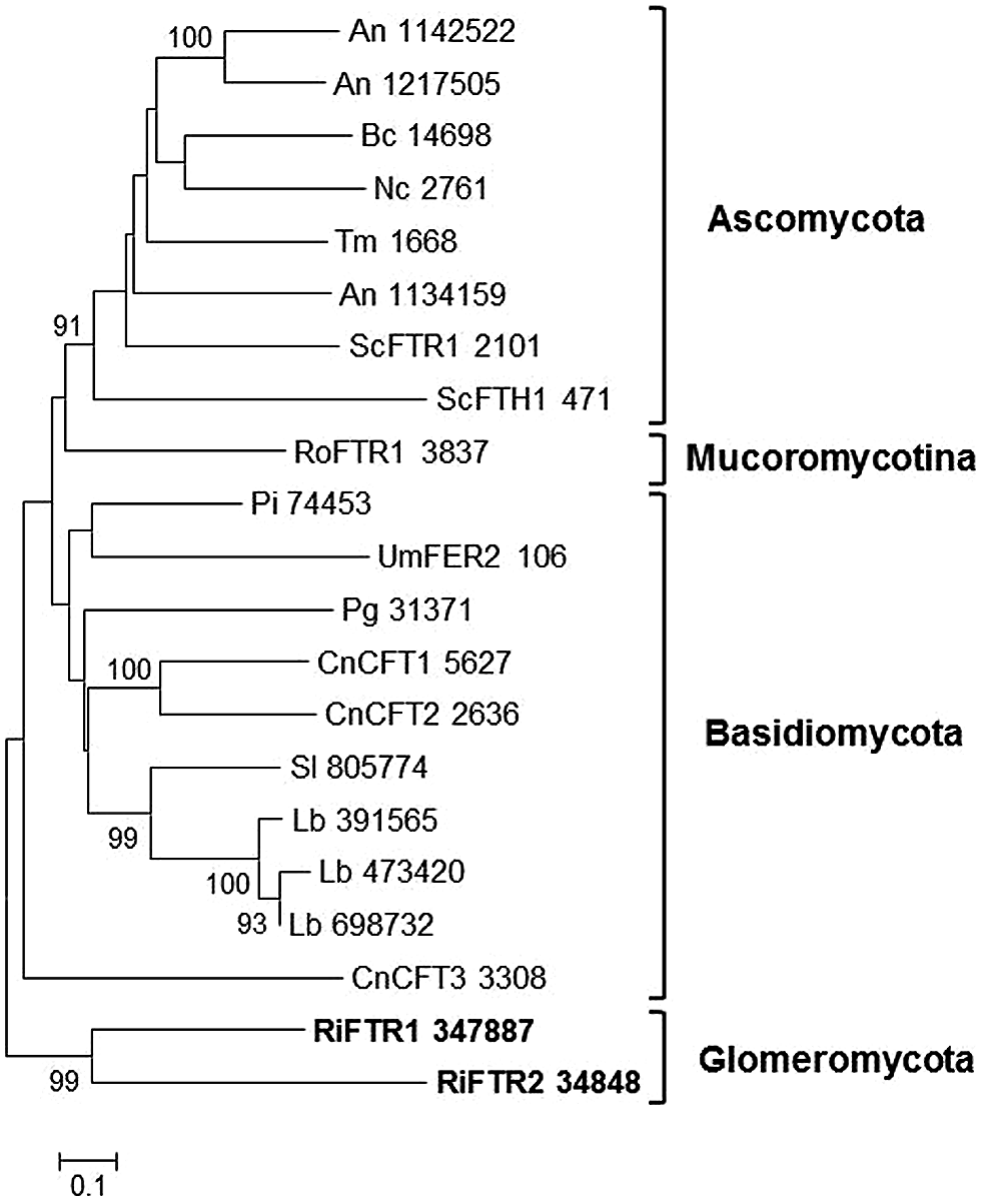
**Phylogenetic relationships of the *Rhizophagus irregularis* iron permeases (FTR) with homologous sequences from selected species representative of the major fungal phyla.** The Neighbor-Joining tree was created with MEGA 6. Protein JGI identification numbers are indicated. *R. irregularis* genes are shown in bold. Organisms: An, *Aspergillus niger*; Bc, *Botrytis cinerea*; Cc, *Coprinopsis cinerea*; Cn, *Cryptococcus neoformans*; Lb, *Laccaria bicolor*; Nc, *Neurospora crassa*; Pi, *Piriformospora indica*; Pg, *Puccinia graminis*; Sc, *Saccharomyces cerevisiae*; Sl, *Suillus luteus*; Tb, *Tuber melanosporum;* Ri, *Rhizophagus irregularis*; Ro, *Rhizopus oryzae*; Um, *Ustilago maydis*. Bootstrap values above 70 and supporting a node used to define a cluster are indicated.

Two putative orthologs of yeast FTR1, named RiFTR1 and RiFTR2, have been found in the *R. irregularis* genome. RiFTR1 and RiFTR2 were more similar to the FTR1 homolog of the Bryophyte *Physcomitrella patens* (43% identity, 66% similarity, and 37% identity, 60% similarity, respectively) than to fungal FTRs. Phylogenetic analyses of the FTR protein sequences of the reference fungi used in this study revealed that RiFTR1 and RiFTR2 clustered together and separated from the other sequences (**Figure 4**). The two *R. irregularis* Fe permeases were predicted to have seven transmembrane helixes and the two REXXE motifs typical of Fe transporters.

Many fungal species, such as *S. cerevisiae*, *C. albicans*, *F. graminearum,* and *C. neoformans* (Urbanowski and Piper, 1999; Fang and Wang, 2002; Park et al., 2006; Han et al., 2012), also possess two or more FTR paralogs. In *S. cerevisiae*, ScFTH1 is located in the vacuole and together with the ferroxidase FET5 exports Fe from the vacuole into the cytosol (Spizzo et al., 1997; Urbanowski and Piper, 1999). However, in *C. neoformans*, which possesses three FTRs, two of them are redundant (Han et al., 2012). Functional characterization of RiFTR1 and RiFTR2 is needed to determine if one of these Fe permeases is involved in Fe mobilization of vacuolar stores or if they are functionally redundant.

Expression analysis of the genes putatively involved in the ferroxidation/permeation pathway in the available transcriptomic data of *R. irregularis* revealed that the two Fe permeases identified in the genome are expressed in germinated spores and mycorrhizal roots (**Table 1**) and that some of the putative multicopper oxidases are expressed in all fungal structures (data not shown), which suggest that the Fe reductive assimilation pathway operates in AM fungi. Interestingly, the Fe permease RiFTR1 was up-regulated (10-fold) during the symbiotic phase of the fungus (**Table 1**). These data suggest that this high-affinity Fe uptake system plays a role not only in Fe uptake from the soil, but also during the biotrophic phase of the fungus. In this respect, it is noteworthy that full virulence of the plant pathogenic fungus *U. maydis* requires a ferroxidation/permeation Fe uptake system (Eichhorn et al., 2006) and it has been postulated that Fe acquisition through the siderophore-mediated pathway is necessary for *Epichloë festucae* to maintain mutualism with perennial ryegrass (Johnson et al., 2013).

The significance of the expression profiles of the Fe uptake systems present in *R. irregularis* is unknown. A recent genome-wide analysis of transcription patterns in defined cell-types of *M. truncatula* roots colonized by *R. irregularis* identified two metal transporters, MtNRAMP1 and MtNRAMP3 likely playing a role in Fe homeostasis, that were induced more than threefold in cortical root cells colonized by arbuscules (Hogekamp and Küster, 2013). Given that in the symbiotic interface enough Fe must be supplied for the metabolism of the plant and the fungus and that excess Fe must be avoided to prevent formation of reactive oxygen species, it is tempting to speculate that maintenance of Fe homeostasis in the symbiotic interface may be essential for the maintenance of a successful symbiosis.

#### Vacuolar iron transport

**FIGURE 5:**
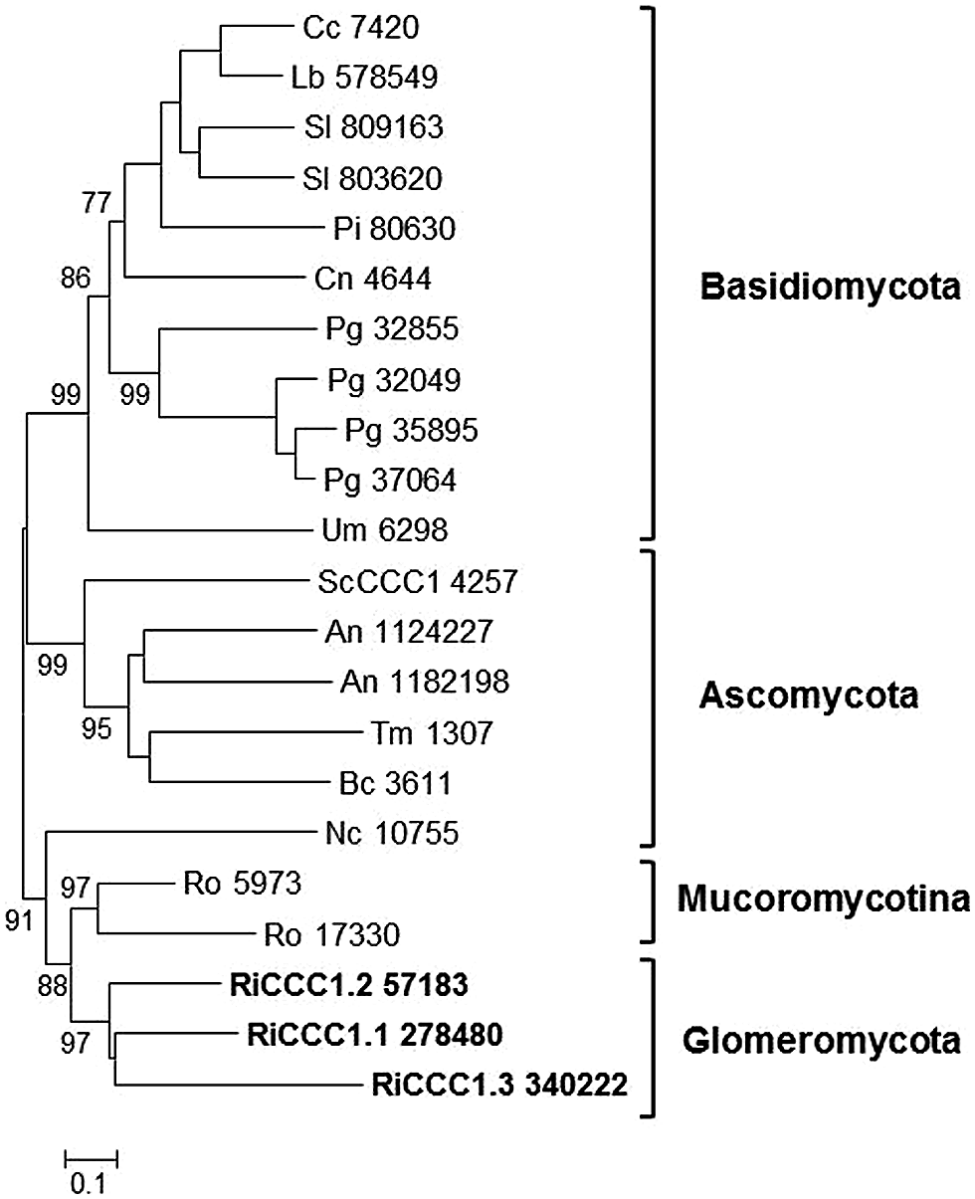
**Phylogenetic relationships of the *Rhizophagus irregularis* vacuolar iron transporters (VIT) with homologous sequences from selected species representative of the major fungal phyla.** The Neighbor-Joining tree was created with MEGA 6. Protein JGI identification numbers are indicated. *R. irregularis* genes are shown in bold. Organisms: An, *Aspergillus niger*; Bc, *Botrytis cinerea*; Cc, *Coprinopsis cinerea*; Cn, *Cryptococcus neoformans*; Lb, *Laccaria bicolor*; Nc, *Neurospora crassa*; Pi, *Piriformospora indica*; Pg, *Puccinia graminis*; Sc, *Saccharomyces cerevisiae*; Sl, *Suillus luteus*; Tb, *Tuber melanosporum;* Ri, *Rhizophagus irregularis*; Ro, *Rhizopus oryzae*; Um, *Ustilago maydis*. Bootstrap values above 70 and supporting a node used to define a cluster are indicated.

Several studies have highlighted the importance of the AM fungal vacuoles for storage and detoxification of heavy metals (Turnau et al., 1993; González-Guerrero et al., 2007; Nayuki et al., 2014). Iron is likely stored in the fungal vacuoles in the ferric form as polyphosphate. In yeast, Fe is loaded into the vacuole by CCC1, a member of the vacuolar iron transporter (VIT) family (Li et al., 2001). Homologs are found in eukaryotes, bacteria, and archaea. Most fungal species encode one CCC1 protein and some others, such as *Aspergillus* and *Rhizopus* species, encode two paralogs (Gsaller et al., 2012). In contrast, three putative paralogs have been found in *R. irregularis*, named RiCCC1.1, RiCCC1.2, and RiCCC1.3. As shown in the phylogenetic tree of fungal VITs, the three putative *R. irregularis* paralogs are closely related and cluster together with the *R. oryzae* homologs, clearly separated from sequences of Ascomycota and Basidiomycota (**Figure 5**). The three paralogs were differentially expressed in the different fungal structures. RiCCC1.1 was the most highly expressed paralog in the ERM. While RiCCC1.2 was down-regulated in mycorrhizal roots, RiCCC1.3 was up-regulated (**Table 1**).

When Fe is low, mobilization of the vacuolar Fe stores should be mediated by a Fe permease/oxidase complex, as it was discussed in the former section, and/or by a homolog of the *S. cerevisiae* NRAMP family member SMF3 that exports Fe from the vacuole into the cytosol (see the NRAMP family section).

### ZINC

In eukaryotes, Zn homeostasis is largely attributed to the coordinated action of two transporter families: the ZIP (zinc-iron permease or ZRT-IRT-like Protein) and the CDF (Cation Diffusion Facilitator) families (Eide, 2006). Only three Zn transporters of the CDF family have been characterized so far in mycorrhizal fungi. The first one was identified in *R. irregularis* (González-Guerrero et al., 2005), the second in *Hebeloma cylindrosporum* (Blaudez and Chalot, 2011) and more recently a new one has been reported in the ericoid fungus *Oidiodendron maius* (Khouja et al., 2013).

#### The zinc-iron permease (ZIP) family

The name of the ZIP family refers to the first members that were functionally characterized, the *S. cerevisiae* Zn transporter ZRT1 and the *Arabidopsis thaliana* Fe transporter IRT1. A key feature of the ZIP family is that, without any yet known exceptions, these proteins transport Zn and/or other metal ion substrates from the extracellular space or organellar lumen into the cytoplasm. ZIP transporters are found at all phylogenetic levels including bacteria, fungi, plants, and mammals (Eide, 2006). Most ZIP proteins have eight predicted transmembrane domains and similar predicted topologies with the N- and C-termini of the protein located on the extracytoplasmic face of the membrane. A histidine-rich region present between transmembrane regions three and four is necessary for Zn selectivity (Nishida et al., 2008).

**FIGURE 6:**
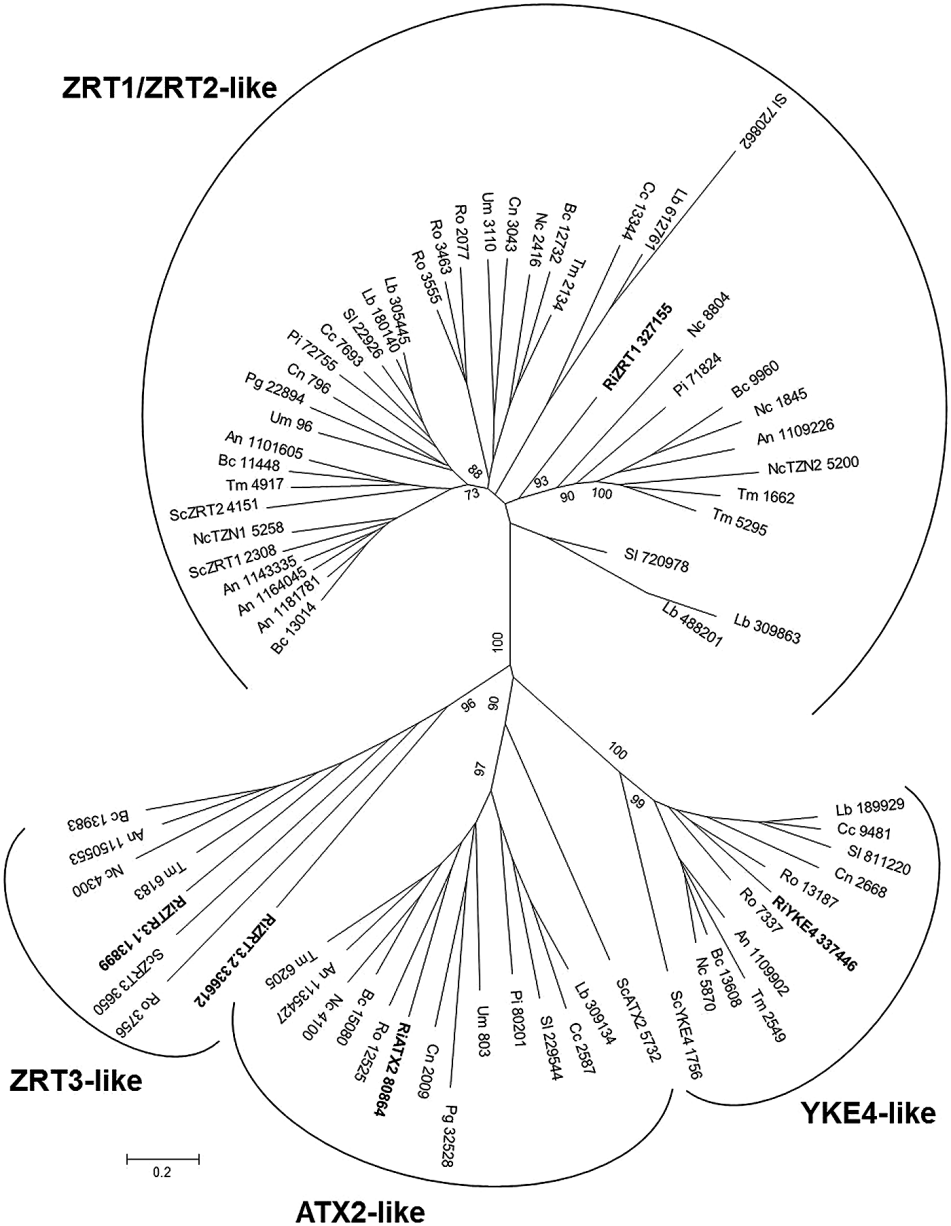
**Phylogenetic relationships of the *Rhizophagus irregularis* zinc-iron permeases (ZIPs) with homologous sequences from selected species representative of the major fungal phyla.** The Neighbor-Joining tree was created with MEGA 6. Protein JGI identification numbers are indicated. *R. irregularis* genes are shown in bold. Organisms: An, *Aspergillus niger*; Bc, *Botrytis cinerea*; Cc, *Coprinopsis cinerea*; Cn, *Cryptococcus neoformans*; Lb, *Laccaria bicolor*; Nc, *Neurospora crassa*; Pi, *Piriformospora indica*; Pg, *Puccinia graminis*; Sc, *Saccharomyces cerevisiae*; Sl, *Suillus luteus*; Tb, *Tuber melanosporum;* Ri, *Rhizophagus irregularis*; Ro, *Rhizopus oryzae*; Um, *Ustilago maydis*. Bootstrap values above 70 and supporting a node used to define a cluster are indicated.

The number of ZIP genes in the genomes of the reference fungi ranges from two to eight (**Table 2**). In *S. cerevisiae,* five ZIP family members have been described: ZRT1, ZRT2, ZRT3, ATX2, and YKE4. The *R. irregularis* ZIP family also includes five candidate genes, which have been named according to their closest yeast orthologs. The fungal ZIP family is divided into four distinct subfamilies. One of the *R. irregularis* paralogs clusters with the plasma membrane high- and low-affinity Zn transporters ZRT1 and ZRT2 of *S. cerevisiae* in the ZRT1/ZRT2-like group (Zhao and Eide, 1996). Two paralogs were grouped in the ZRT3-like cluster and are closely related to the *S. cerevisiae* ZRT3, which mediates Zn release from the vacuole to the cytosol (Simm et al., 2007). The ATX2-like and YKE-like subfamilies including, respectively, the yeast ATX2 protein involved in Mn trafficking (Lin and Culotta, 1996) and the bidirectional Zn transporter YKE4 (Kumánovics et al., 2006), also comprised one *R. irregularis* gene each (**Figure 6**). ZIP member distribution in the phylogenetic tree was not related to organism taxonomy, but rather to substrate specificity or subcellular location suggesting ancient duplication events followed by subfunctionalization in a common ancestor.

Expression analyses revealed that all the *R. irregularis* paralogs were expressed in all the fungal structures tested. RiZRT1 was highly up-regulated in mycorrhizal roots relative to the expression levels detected in germinated spores (420-fold) and in the ERM (eightfold). The two homologs of the yeast vacuolar Zn transporter, RiZTR3.1 and RiZTR3.2, were also up-regulated (threefold) in mycorrhizal roots (**Table 1**). These expression data suggest that the fungus takes up Zn from the apoplast of the symbiotic interface and that a mobilization of the vacuolar Zn stores occurs in the IRM. These data reinforce our hypothesis that AMF must maintain proper metal homeostasis for colonization and survival in the host roots.

#### The cation diffusion facilitator (CDF) family

**FIGURE 7:**
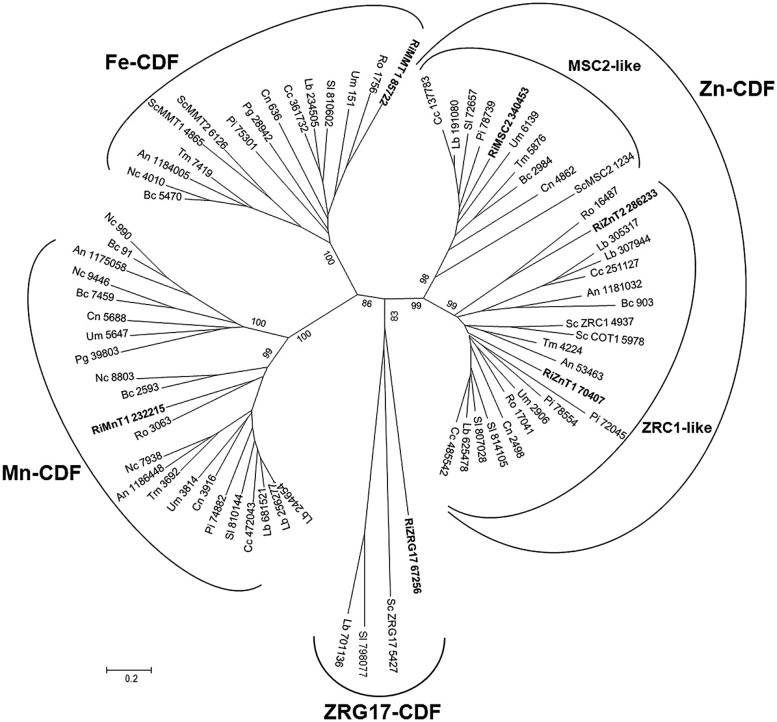
**Phylogenetic relationships of the *Rhizophagus irregularis* cation diffusion facilitators (CDFs) with homologous sequences from selected species representative of the major fungal phyla.** The Neighbor-Joining tree was created with MEGA 6. Protein JGI identification numbers are indicated. *R. irregularis* genes are shown in bold. Organisms: An, *Aspergillus niger*; Bc, *Botrytis cinerea*; Cc, *Coprinopsis cinerea*; Cn, *Cryptococcus neoformans*; Lb, *Laccaria bicolor*; Nc, *Neurospora crassa*; Pi, *Piriformospora indica*; Pg, *Puccinia graminis*; Sc, *Saccharomyces cerevisiae*; Sl, *Suillus luteus*; Tb, *Tuber melanosporum;* Ri, *Rhizophagus irregularis*; Ro, *Rhizopus oryzae*; Um, *Ustilago maydis*. Bootstrap values above 70 and supporting a node used to define a cluster are indicated.

The key feature of this family is that they transport Zn and/or other metal ions from the cytoplasm into the lumen of intracellular organelles or to the outside of the cell. Thus, CDF proteins work in opposition to the ZIP transporters. CDF transporters are also found at all phylogenetic levels. Most members of this family have six predicted transmembrane domains with the N- and C-termini predicted to be cytoplasmic. A notable exception to this rule is the yeast MSC2 protein that forms a heteromeric CDF complex with ZRG17 to transport Zn into compartments of the secretory pathway. Like the ZIP proteins, many CDF family members have histidine rich motifs, in this case usually in the cytoplasmic loop between transmembrane domains 4 and 5. The majority of CDF family members are classified into three groups, each containing characterized members that share the same specificity toward the principally transported metal, Zn, Fe/Zn, or Mn. An additional group is the ZRG17-like subfamily, which is very distant from the Zn-CDF but with similar biochemical characteristics (Montanini et al., 2007). Six genes putatively encoding CDFs were identified in *R. irregularis,* which have been named according to their closest yeast orthologs. Three were included in the Zn-CDF subfamily, two in the ZRC1-like cluster and one in the MSC2-like cluster (**Figure 7**). The ZRC1-like cluster comprises the yeast vacuolar Zn transporters ZRC1 and COT1 (MacDiarmid et al., 2002) and the *R. irregularis* CDF GiZnT1 (González-Guerrero et al., 2005, renamed here as RiZnT1). These transporters mediate Zn uptake into the vacuole and are involved in Zn tolerance. Members of the MSC2-cluster also transport Zn, but into the endoplasmic reticulum. Another *R. irregularis* CDF, named RiMMT1, was grouped together with the *S. cerevisiae* mitochondrial Fe transporters MMT1 and MMT2 in the Fe-CDF subfamily and it is likely to mediate the transport of Fe. The other two homologs found, RiMnT1, and RiZRG17, were grouped in the Mn-CDF and ZRG17-like subfamilies, respectively, and are proposed to be involved in the transport of Mn and Zn (Montanini et al., 2007; Diss et al., 2011). Although the metal specificity of the newly identified CDF transporters of *R. irregularis* has been inferred from their distribution in the phylogenetic tree, an exhaustive functional characterization of these transporters is needed to confirm the transport processes mediated by the different isoforms.

Expression analyses of the CDF family members revealed that the different orthologs are expressed in all fungal structures analyzed, except RiZnT1 that was expressed at very low levels in germinated spores (**Table 1**).

### THE NATURAL RESISTANCE-ASSOCIATED MACROPHAGE PROTEINS (NRAMP) FAMILY OF DIVALENT METAL TRANSPORTERS

The NRAMP family constitutes a class of divalent metal transporters that are highly conserved from bacteria to mammals. These transporters use the transmembrane proton gradient to facilitate transport of a broad range of divalent cations toward the cytosol. *S. cerevisiae* has three homologs of this family in its genome, SMF1, SMF2, and SMF3. SMF1, and SMF2 mainly transport Mn, although SMF1 also transports Fe. While SMF1 operates at the plasma membrane in the uptake of either Mn or Fe (Chen et al., 1999; Portnoy et al., 2000), SMF2 is localized on membranes of intracellular Golgi vesicles, being involved in transport of Mn out of the vesicles (Reddi et al., 2009). SMF3 exports iron from the vacuole to the cytosol (Diffels et al., 2006), and together with CCC1, is responsible for Fe homeostasis in this organelle.

**FIGURE 8:**
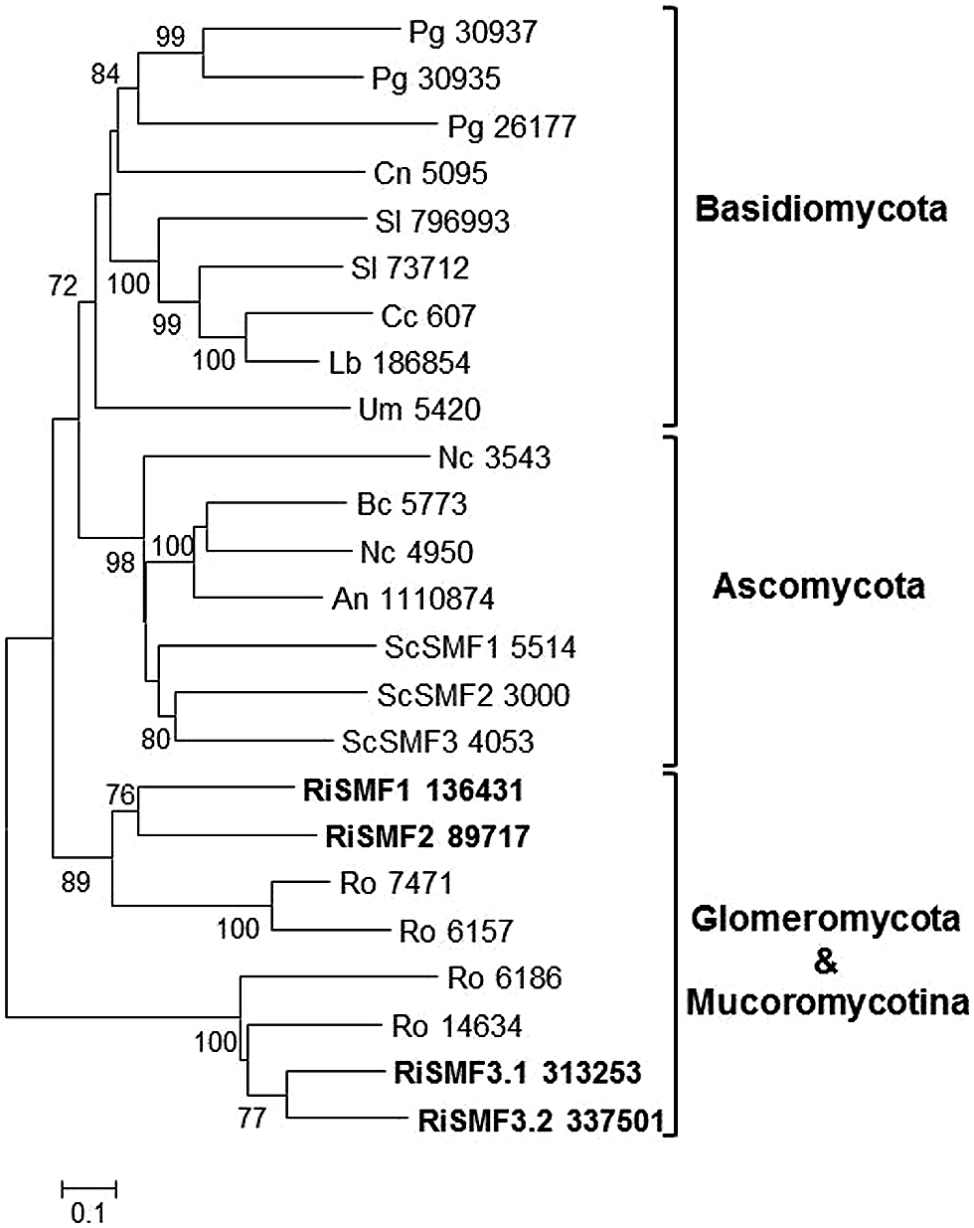
**Phylogenetic relationships of the *Rhizophagus irregularis* natural resistance-associated macrophage proteins (NRAMPs) with homologous sequences from selected species representative of the major fungal phyla.** The Neighbor-Joining tree was created with MEGA 6. Protein JGI identification numbers are indicated. *R. irregularis* genes are shown in bold. Organisms: An, *Aspergillus niger*; Bc, *Botrytis cinerea*; Cc, *Coprinopsis cinerea*; Cn, *Cryptococcus neoformans*; Lb, *Laccaria bicolor*; Nc, *Neurospora crassa*; Pi, *Piriformospora indica*; Pg, *Puccinia graminis*; Sc, *Saccharomyces cerevisiae*; Sl, *Suillus luteus*; Tb, *Tuber melanosporum;* Ri, *Rhizophagus irregularis*; Ro, *Rhizopus oryzae*; Um, *Ustilago maydis*. Bootstrap values above 70 and supporting a node used to define a cluster are indicated.

Searches in the *R. irregularis* genome led to the identification of four putative NRAMP homologs, all of them having the signature sequence DPGN. The sequences from Basidiomycota clearly separated from those of Ascomycota in the phylogenetic tree. The *R. oryzae* and *R. irregularis* homologs were grouped together in two different clades (**Figure 8**). Analysis of the available gene expression profiles of *R. irregularis* revealed that SMF1 and SMF3.1 are expressed in all fungal structures and that RiSMF1 and RiSMF2 are expressed at very low levels in germinated spores (Tisserant et al., 2012).

## CONCLUSION

**FIGURE 9:**
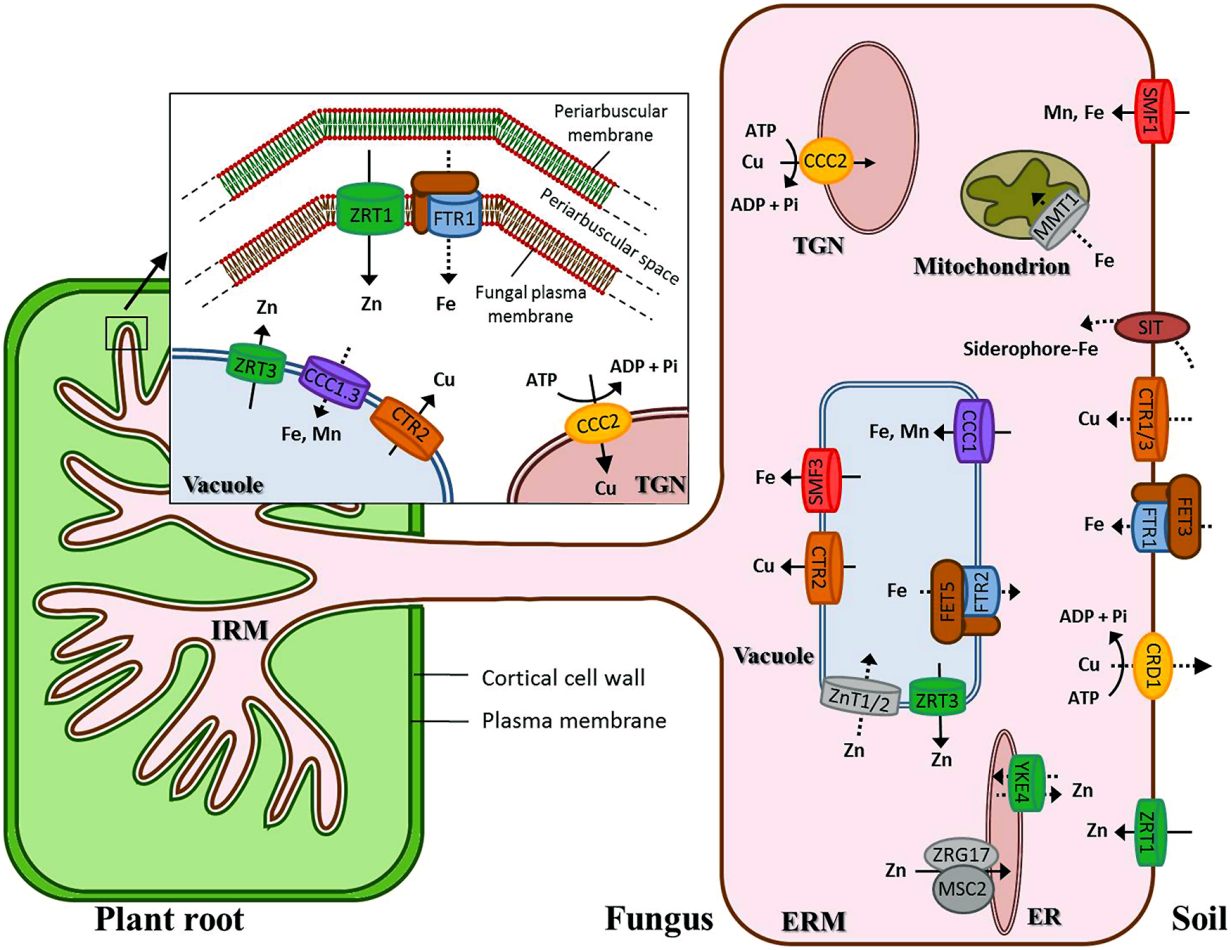
**Schematic representation of the putative Cu, Fe, and Zn transport systems in *Rhizophagus irregularis*.** Transcripts for all these transporters have been detected in symbiotic roots and germinated spores. Discontinuous arrows refer to transporters whose transcript levels in the ERM have not been determined yet. Transcripts up-regulated by more than 2.5-fold in the symbiotic roots are shown in the intraradical mycelium (IRM), except ZnT1 that was expressed at very low levels in germinated spores and SMF1 that was only up-regulated in the symbiotic roots relative to germinated spores but not to the ERM. Putative Cu transporters (CTR) are in dark orange; P_1B_ -or Cu-ATPases (CCC2) in light orange; the oxidase-dependent Fe^2+^transporter (OFeT) family members in brown (ferroxidases) and blue (permeases); zinc-iron permeases (ZIPs) in green; cation diffusion facilitators (CDFs) in gray, NRAMPs in red and VITs in purple. ER, endoplasmic reticulum; TGN, trans-Golgi network.

The present analysis aimed at establishing a repertoire of candidate genes that represent the genetic potential for transport of Fe, Cu, and Zn in *R. irregularis*. We have revealed the presence of at least 30 genes encoding putative transition metal transporters, showing all of them detectable transcript levels in the fungal structures analyzed. **Figure 9** summarizes the candidate genes identified in this genomic survey. The sequences and expression information reported herein will be useful for further investigation of the roles of these transport proteins in Cu, Fe, and Zn homeostasis in AMF and in the symbiosis. A comprehensive physiological analysis of the current dataset needs detailed characterization of the encoded proteins. However, we would like to highlight two features that stood out in this *in silico* analysis: (i) expansion of some families of metal transporters, specifically of Cu-ATPases, VITs, and NRAMPs, and (ii) up-regulation of a certain number of genes putatively encoding transport proteins mediating the influx of Fe/Zn (RiFTR1, RiZRT1) and the mobilization of the vacuolar Cu/Zn stores (RiCTR2, RiZRT3) in the intraradical phase of the fungus. Since these transporters are unlikely to be involved in metal transfer to the plant, they should play a role in maintaining Fe, Cu, and Zn homeostasis in the IRM. Based on these observations we speculate that metal homeostasis in the places of close interactions between the plant and the fungus is needed for symbiotic development, as it has been shown in the mutualistic symbioses formed between *Epichloë* endophytes and the Poaceae. The challenge now is to functionally characterize these transporters and to identify their location and roles in the symbiosis.

## AUTHOR CONTRIBUTIONS

The work presented here was carried out in collaboration between all authors. Elisabeth Tamayo and Tamara Gómez-Gallego performed the majority of the *in silico* analyses. Concepción Azcón-Aguilar and Nuria Ferrol defined the research theme. Nuria Ferrol coordinated the project and Elisabeth Tamayo and Nuria Ferrol wrote the manuscript. All authors have contributed to, seen and approved the manuscript.

## Conflict of Interest Statement

All authors declare that the research was conducted in the absence of any commercial or financial relationships that could be construed as a potential conflict of interest.

## Footnotes

1 http://www.tcdb.org/

2 http://www.membranetransport.org/

3 http://genome.jgi-psf.org/Gloin1

4 http://genome.jgi-psf.org/Lacbi2

5 http://genome.jgi.doe.gov/Tubme1

6 http://genome.jgi.doe.gov/Rhior3

7 http://www.ncbi.nlm.nih.gov/cdd

8 http://www.cbs.dtu.dk/services/TMHMM/

9 http://smart.embl-heidelberg.de/

10 http://www.ebi.ac.uk/Tools/msa/clustalw2/
